# The complete mitochondrial genome of *Arhopalus unicolor* (Coleoptera: Cerambycidae)

**DOI:** 10.1080/23802359.2021.1875923

**Published:** 2021-02-13

**Authors:** Jianrong Wen, Yuting Li, Zuohua Huang, Jiayi Ma, Shaozhen Wang, Songqing Wu

**Affiliations:** a College of Forestry, Fujian Agriculture and Forestry University, Fuzhou, China; b Xiamen Customs Technical Center, Xiamen, China; c Key Laboratory of Integrated Pest Management in Ecological Forests, Fujian Province University, Fujian Agriculture and Forestry University, Fuzhou, China

**Keywords:** Complete mitochondrial genome, Arhopalus unicolor, phylogenetic analysis

## Abstract

In this study, the complete mitochondrial genome of *Arhopalus unicolor* was first sequenced. The length of the complete mitochondria genome of *A. unicolor* was 15,760 bp with 19.0% GC content, including 40.5% A, 10.8% C, 8.2% G and 40.5% T. There were 13 protein-coding genes (CDS), 22 transfer RNA genes (tRNA), 2 ribosomal RNA genes (rRNA), and an AT-rich region. Phylogenetic analysis showed that *A. unicolor* was closely related to *Spondylis buprestoides*. This study provides useful genetic information for subsequent prevention of *A. unicolor*.

*Arhopalus unicolor* is the main trunk borer of pine trees, and the larvae can serious damage to xylem and phloem tissues (Li et al. 2006). *A. unicolor* can also carry *Bursaphelenchus xylophilus*, which causes pine wilt disease, killing pine trees and bringing huge economic and ecological losses (Li et al. 2006). Therefore, it is particularly important for the control of *A. unicolor*. In order to understand the genetic and evolutionary information of the species, so as to better carry out the prevention and treatment work, we determined the complete mitochondria genome of *A. unicolor* from China in this study.

The *A. unicolor* adults were collected from Minghou, Fujian Province, China (119°19′5.18″E, 25°52′25.3″N) by the traps. The specimens were stored in the Key Laboratory of Integrated Pest Management in Ecological Forests, Fujian Province University, Fujian Agriculture and Forestry University (TN-202008). The total DNA was extracted by the TruSeq DNA Sample Preparation Kit (Vanzyme, Nanjing, China) and purified by the QIAquick Gel Extraction Kit (Qiagen GmbH, Germany). The mitochondrial genome was sequenced by the Illumina Hiseq 2500 at Genesky Biotechnologies Inc. (Shanghai, China). In all, 75,829,218 clean reads were obtained through the quality analysis and filtration. Then, these clean reads were assembled by MtioZ and metaSPAdes software (Nurk et al. 2017). The assembly mitogenome was annotated by GeSeq (Tillich et al. 2017), and manually adjusted in Geneious based on the reference sequence of *Xylotrechus grayii* (GenBank accession no. KM112084) (Kearse et al. 2012). The complete mitogenome sequence of *A. unicolor* has been submitted to NCBI Genbank with accession number MW067122. The length of the complete mitochondria genome of *A. unicolor* was 15,760 bp with 40.5% A, 10.8% C, 8.2% G, 40.5% T. The GC content of the complete genome was 19.0%, and there were 13 protein-coding genes, 22 tRNAs, 2 rRNAs, and an AT-rich region.

To further investigate the phylogenetic position of *A. unicolor*, according to the genome sequence of *A. unicolor*, the sequence alignment was performed with the MAFFT (Katoh and Standley 2013). And MEGA 7 was used to construct the corresponding neighbor-joining phylogenetic tree with 15 different species of Coleoptera, following by calculating the bootstrap values with 1000 replications (Kumar et al. 2016). The result showed that *A. unicolor* and *S. buprestoides* were clustered together, and sister to *Cortodera humeralis* (Figure 1). The complete mitochondrial genome of *A. unicolor* will provide useful information to study the genetic evolution of *A. unicolor* and control them, as well as in insects of *Arhopalus.*

**Figure 1.**
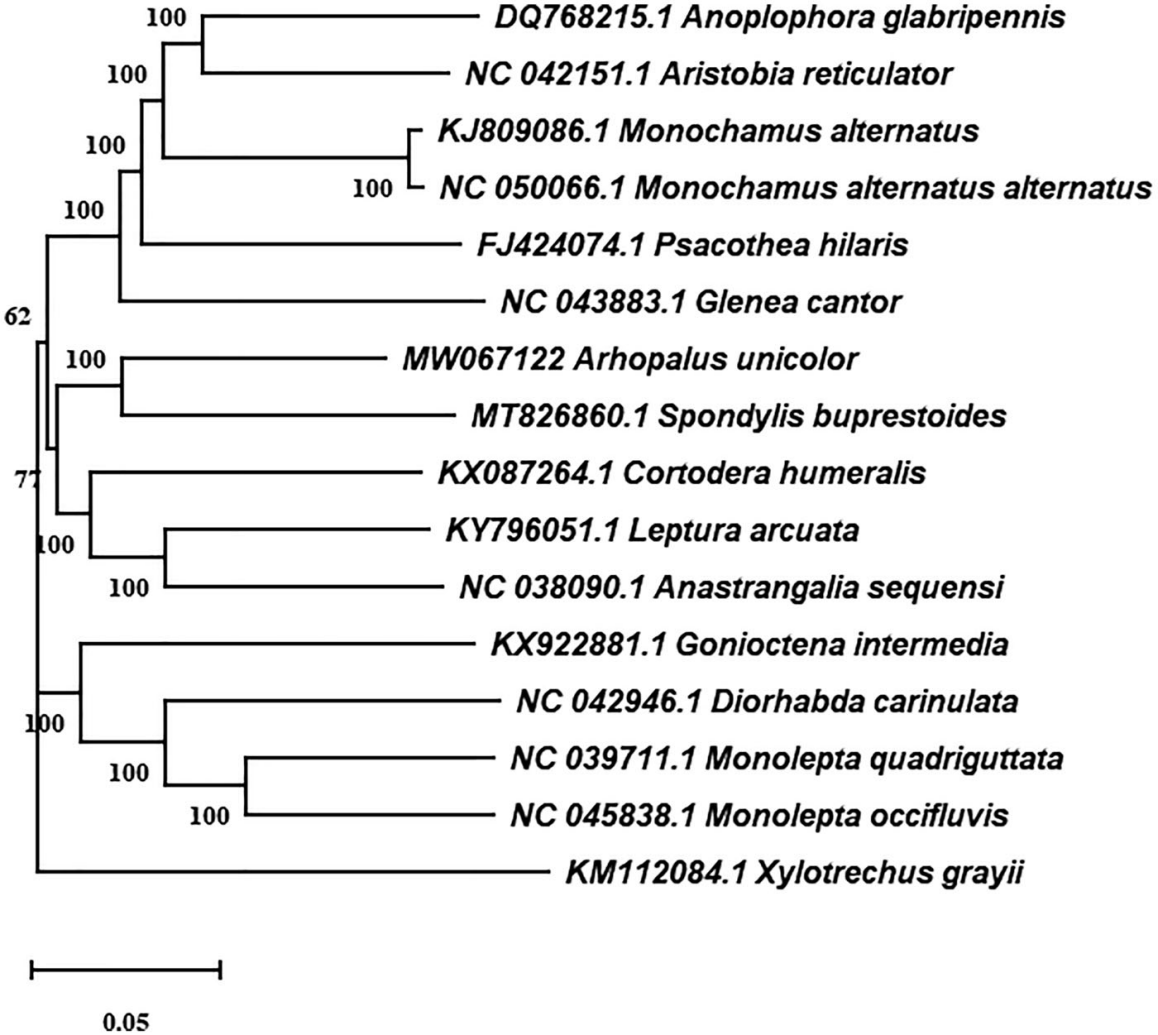
Neighbor-joining tree of the *Arhopalus unicolor* and related 15 different species of Coleoptera based on the genome sequence. Numbers labeled on the branch are bootstrap values.

## Data Availability

The data that support the findings of this study are openly available in GenBank of NCBI at https://www.ncbi.nlm.nih.gov, reference number MW067122.
